# Supplementary material for: Resolving the full spectrum of human genome variation using Linked-Reads
Source: Genome Res. 2019 Apr;29(4):635–45. doi: 10.1101/gr.234443.118 (PMC6442396; doi:10.1101/gr.234443.118)
Supplement: Supplemental Material [file supp_gr.234443.118_Supplemental_longranger-2.2.zip › longranger-2.2.tar.bz2!/lr2.2-public/lib/stan/cmdstan/stan/doc/api/html/ordered__logistic_8hpp.html]

Stan: src/stan/prob/distributions/univariate/discrete/ordered\_logistic.hpp File Reference


|  |  |
| --- | --- |
|  | Stan  2.5.0  probability, sampling & optimization |


- Main Page
- Related Pages
- Namespaces
- Classes
- Files

- File List
- File Members

All Classes Namespaces Files Functions Variables Typedefs Enumerations Enumerator Friends Macros Pages

- src
- stan
- prob
- distributions
- univariate
- discrete

Namespaces |
Functions

ordered\_logistic.hpp File Reference

`#include <boost/random/uniform_01.hpp>`  
`#include <boost/random/variate_generator.hpp>`  
`#include <stan/prob/distributions/multivariate/discrete/categorical.hpp>`  
`#include <stan/prob/traits.hpp>`  
`#include <stan/math/error_handling.hpp>`  
`#include <stan/math/functions/inv_logit.hpp>`  
`#include <stan/math/functions/log1m.hpp>`  
`#include <stan/math/functions/log1m_exp.hpp>`  
`#include <stan/math/functions/log1p_exp.hpp>`  
`#include <stan/math/matrix_error_handling.hpp>`  
`#include <stan/prob/constants.hpp>`

Go to the source code of this file.

|  |  |
| --- | --- |
| Namespaces | |
|  | stan |
|  | Probability, optimization and sampling library. |
|  | |
|  | stan::prob |
|  | Templated probability distributions. |
|  | |

|  |  |
| --- | --- |
| Functions | |
| template<typename T > | |
| T | stan::prob::log\_inv\_logit\_diff (const T &alpha, const T &beta) |
|  | |
| template<bool propto, typename T\_lambda , typename T\_cut > | |
| boost::math::tools::promote\_args  < T\_lambda, T\_cut >::type | stan::prob::ordered\_logistic\_log (int y, const T\_lambda &lambda, const Eigen::Matrix< T\_cut, Eigen::Dynamic, 1 > &c) |
|  | Returns the (natural) log probability of the specified integer outcome given the continuous location and specified cutpoints in an ordered logistic model. More... |
|  | |
| template<typename T\_lambda , typename T\_cut > | |
| boost::math::tools::promote\_args  < T\_lambda, T\_cut >::type | stan::prob::ordered\_logistic\_log (int y, const T\_lambda &lambda, const Eigen::Matrix< T\_cut, Eigen::Dynamic, 1 > &c) |
|  | |
| template<class RNG > | |
| int | stan::prob::ordered\_logistic\_rng (const double eta, const Eigen::Matrix< double, Eigen::Dynamic, 1 > &c, RNG &rng) |
|  | |


---

[ Stan Home Page ]

*© 2011–2014,
Stan Development Team.*
